# Risk factors associated with syphilis among patients at a sexual health center in Brazil: A retrospective study

**DOI:** 10.1016/j.bjid.2025.104558

**Published:** 2025-06-28

**Authors:** Cristhiane Campos Marques, Fabiana Nunes de Carvalho Mariz, Thaisa Campos Marques, Juliana Tessari Dias Rohr, Berenice Moreira, Paula Beatriz Santiago, Nathália Araujo Varela da Costa, Juliana Marques de Oliveira, Otávio Toledo Nóbrega, Carla Nunes de Araújo

**Affiliations:** a Universidade de Brasília, Faculdade de Medicina, Programa de Pós-Graduação em Ciências Médicas, Brasília, DF, Brazil; b Universidade de Rio Verde, Rio Verde, GO, Brazil; c Centro de Testagem e Aconselhamento, Rio Verde, GO, Brazil; d Universidade Católica de Brasília, Brasília, DF, Brazil; e University of California, Davis, USA; f Universidade de Brasília, Departamento de Biologia Celular, Laboratório de Interação Patógeno-Hospedeiro, Brasília, DF, Brazil; g Universidade de Brasília, Faculdade de Ciências e Tecnologias em Saúde, Centro Metropolitano Ceilândia Sul, Brasília, DF, Brazil

**Keywords:** STI, *Treponema pallidum*, Men who have sex with men, Sexual behavior, Drug use

## Abstract

Syphilis has reemerged worldwide, and knowledge about at-risk populations can assist in disease prevention and control. This study aimed to estimate the prevalence of syphilis among attendees at a Brazilian sexual health center and explore the association between sociodemographic and sexual behavioral factors with syphilis infection. A cross-sectional study was conducted at a Counselling and Testing Center (CTC) in Goiás, Brazil. We collected data from January to December 2018 to conduct a population-based study. Of 3526 patient records included in the analysis, 344 (9.8 %) belonged to participants who had syphilis, mainly at the age of 20 to 39 years old (63.1 %, *p* = 0.0683) and not married (62.1 %, *p* = 0.0042). Individuals who reported having homosexual relationships (Odds Ratio [OR = 2.93], *p* < 0.0001), previous sexually transmitted infections (OR = 2.36, *p* < 0.0001), and use of drugs (OR = 1.29, *p* < 0.0001) were more frequently diagnosed with the disease. Among patients with syphilis, MSM had a higher HIV co-infection rate (OR = 2.37, *p* = 0.0195) and also higher co-infection rates with other previous STIs (OR = 2.84, *p* = 0.00883). A high prevalence of syphilis among patients who attended the CTC in Goiás was revealed. Effective control of syphilis among populations at higher risk needs to be addressed to achieve disease control in Brazil.

## Introduction

Sexually Transmitted Infections (STIs) are a significant public health concern worldwide. Every day, over 1 million curable STIs are acquired.^1^ Different bacteria, viruses, and parasites can be transmitted sexually by an infectious partner. Among them is the Gram-negative spirochete *Treponema pallidum* that causes syphilis. As few as ten spirochetes can cause the disease after penetrating the mucous membranes or damaged skin.^2^ *T pallidum* adheres to epithelial cells or extracellular matrix components,^3^ replicating and disseminating through the lymphatic and blood circulatory systems, thereby rapidly establishing a systemic infection.^4^ Syphilis can also be transmitted vertically during pregnancy.^2^ After malaria, congenital syphilis is the second leading cause of preventable stillbirths worldwide.^5^ Syphilis clinical manifestations are variable and result from the inflammatory process elicited by pathogen replication within tissues, causing neurological, cardiovascular, and dermatological disease.^2^ Characteristically, the disease follows a course of four clinical stages over approximately ten years.^6^

The global incidence of syphilis in men and women aged 15‒49 years was 6.3 million in 2016. The prevalence and incidence rates vary according to the socio-demographic region.^1^ The incidence rate of acquired syphilis in Brazil reached 60 cases per 100,000 individuals by 2017, with an increase of 32 % in registered syphilis cases compared with the previous year and at US$ 3 million/year cost.^7^ High-risk groups are young black women and men, especially Men who have Sex with Men (MSM), drug addicts, and female sex workers.^8^^,^^9^

There is no vaccine against syphilis,^6^ but the disease is curable by the administration of penicillin.^10^ Effective control relies on different strategies. The most important is early detection and prompt treatment of infected people and their partners. Other preventive methods are latex condom use, prevention campaigns, comprehensive sexuality education, counseling, and notification.^6^

It is a challenge to establish an appropriate framework to monitor at-risk syphilis communities. Previous studies have shown that screening sexual and social practices are essential to identify at-risk populations. Emphasis should be placed on data related to intercourse regularity, sexual partners, and preferences, as well as the pattern of drug and condom use and HIV/STI history, among others.^11^, ^12^, ^13^

The analysis of the regional burden of syphilis and the associated risk behaviors offers valuable insights for policymakers to delineate health programs and strategies to help people adopt attitudes that can reduce their risk for syphilis and other STIs, improving their health and well-being. In this context, sexual health centers such as the Counselling and Testing Centers (CTC) in Brazil are fundamental sources of information on the sociodemographic profile and lifestyle of patients with STIs. Here, we aimed to assess the prevalence of syphilis at the regional level among attendees at a CTC in Goiás, Brazil, and to explore sociodemographic traits and sexual behavior as risk factors for syphilis.

## Methods

### Study design

We conducted a cross-sectional study using an associative, retrospective approach at a CTC in Goiás, Brazil, from January to December 2018. CTC is a public sexual health center that offers attendees clinical consultations and advisory services provided by healthcare professionals. We obtained clinical records to collect patient information (including demographics, lifestyle, and laboratory data). Each clinical record corresponds to one CTC patient who attended during the survey.

### Data collection

All clinical records during the study period were eligible. The sociodemographic characteristics included gender, age, marital status, race, level of education, and employment. The main clinical traits raised were previous STI, HIV status, sexual practices (e.g., type of sexual partnership, number of partners, condom use with steady or casual partners), and use of licit and illicit drugs (alcohol, cigarette/cigar, marijuana, cocaine, crack, heroin, amphetamine, and others) in the last 12 months.

In this study, we defined cases of syphilis as CTC attendees with non-treponemal and treponemal reagent tests independent of treatment and symptoms.

### Statistical analysis

Extracted data from the records were transferred to Excel sheets (Microsoft, Redmond, WA, USA), and all analyses were performed using R, version 3.6.1 (R Core Team, Vienna, Austria). A p-value of < 0.05 was considered statistically significant. The data are presented as the mean ± standard deviation for continuous variables and as absolute counts or percentages for categorical variables.

We used the chi-square test to assess associations between categorical traits. We also investigated factors that affect the likelihood of syphilis, such as age, sex, ethnicity, marital status, race/color, formal schooling years, occupation, previous STI, pattern of drug use, condom use, number and steadiness of partners, and homosexual behavior, using a generalized linear model of the binomial family or a logit binding function.

The models were progressively simplified by removing each variable individually to assess their significance. Likelihood Ratio Tests (LRTs) were used, and the change in deviance was evaluated using a chi-square approximation. The simplified model was maintained whenever the removal of a variable did not statistically affect the model fit.

We applied Wald tests to investigate differences between categorical variable categories. We used the binom.confint function in the binom R package to calculate the confidence interval of binomial probability presented in the graphs.

This study was approved by the Research Ethics Committee of the University of Rio Verde (3.824.310).

## Results

We identified 3526 clinical records in the CTC in Goiás from January to December 2018, 344 (9.8 %) of which were from individuals who had syphilis. Of those, 20 (0.6 %) were from individuals with a serological scar. The characteristics of the study population are reported in Table 1. In general, 53 % (*n* = 1867) were men, 57.8 % (*n* = 2038) were 20‒39 years old, 46.9 % (*n* = 1444) were married, 56.9 % (*n* = 2005) self-declared as brown, and 55.5 % (*n* = 1765) were formally employed. The proportion of participants with 12 years or more of schooling in the study population was 35.6 % (*n* = 1035). In terms of sexual health, 83.2 % (*n* = 2168) reported no previous STI diagnosis. The proportion of HIV-reagent was 4.6 % (*n* = 161) in the study population.

**Table 1:** Sociodemographic, sexual, and behavioral characteristics of patients who attended the CTC sexual health center in Goiás, Brazil, 2018.

Variables	Total, n ( %)	Syphilis, n ( %)	p-value^a^	OR (95 % CI)
Total patients	3526 (100 %)	344 (9.76 %)		
Sociodemographic				
*Gender*	3526	344	0.0093	
Men	1867 (52.95 %)	198 (57.56 %)		1.47 (1.095–1.959)
Women	1659 (47.05 %)	146 (42.44 %)	Ref	1.000
*Age*	3526	344	0.0683	
13–19	321 (9.10 %)	18 (5.23 %)	Ref	1.000
20–29	1134 (32.16 %)	126 (36.63 %)	0.0117	2.11 (1.1180–3.756)
30–39	904 (25.64 %)	91 (26.45 %)	0.0385	1.93 (1.035–3.583)
40–49	550 (15.60 %)	48 (13.95 %)	0.1298	
≥ 50	617 (17.50 %)	61 (17.63 %)	0.2055	
*Marital status*	3077	319^b^	0.0042	
Married	1444 (46.93 %)	121 (37.93 %)	Ref	1.000
Single	1383 (44.95 %)	162 (50.78 %)	0.0031	1.45 (1.132–1.859)
Divorced	161 (5.23 %)	23 (7.21 %)	0.0128	1.82 (1.129–2.942)
Widower	89 (2.89 %)	13 (4.08 %)	0.0435	1.87 (1.009–3.466)
*Race/color*	3526	344	0.2238	
White	519 (14.72 %)	42 (12.21 %)		
Black	195 (5.53 %)	23 (6.69 %)		
Yellow	14 (0.40 %)	2 (0.58 %)		
Brown	2005 (56.86 %)	225 (65.41 %)		
Ignored	793 (22.49 %)	52 (15.12 %)		
*Schooling (years)*	2908	312^b^	0.0017	
None	58 (1.99 %)	11 (3.53 %)	Ref	
1–3	250 (8.60 %)	28 (8.97 %)	0.1068	
4–7	623 (21.42 %)	82 (26.28 %)	0.2076	
8–11	942 (32.39 %)	103 (33.01 %)	0.0447	0.49 (0.247–0.983)
≥12	1035(35.59 %)	88 (28.21 %)	0.0085	0.4 (0.197–0.788)
*Employment*	3182	307^b^	0.0002	
Retired	121 (3.80 %)	15 (4.89 %)	0.5914	
Truck driver	164 (5.15 %)	17 (5.54 %)	<0.0001	Ref
Unemployed	122 (3.83 %)	13 (4.23 %)	0.9182	
Housewives	398 (12.51 %)	56 (18.24 %)	0.2350	
Student	421 (13.23 %)	30 (9.77 %)	0.663	
Healthcare Professional	181 (5.69 %)	2 (0.65 %)	<0.0001	0.1 (0.022–0.425)
Sex worker	5 (0.16 %)	0	0.4478	
Employed	1765 (55.47 %)	173 (56.35 %)	0.8166	
Does not work	5 (0.16 %)	1 (0.33 %)	0.4915	
Sexual characteristics				
*Previous STI*	2607	293^b^	<0.0001	
Yes	439 (16.84 %)	87 (29.69 %)		2.36 (1.782–3.092)
No	2168 (83.16 %)	206 (70.31 %)	Ref	1.000
*HIV*	3526	344	<0.0001	
Positive	161 (4.57 %)	36 (10.47 %)		0.35 (0.237–0.516)
Negative	3365 (95.43 %)	308 (89.53 %)	Ref	1.000
*Relationship*	2363 (100 %)	274^b^	<0.0001	
Heterosexual	2165 (91.62 %)	224 (81.75 %)	Ref	1.000
Homosexual	198 (8.38 %)	50 (18.25 %)		2.93 (2.050–4.132)
*Number of partners*	2365(100 %)	274^b^	<0.0001	
1	1621 (68.54 %)	154 (56.20 %)		0.55 (0.423–0.706)
≥2	744 (31.46 %)	120 (43.80 %)	Ref	1.000
*Condom use with steady partners*	1979	211	0.0004	
Did not use condom	1231 (62.20 %)	109 (51.66 %)	Ref	1.000
Not always with condom	373 (18.85 %)	36 (17.06 %)	0.6318	1.10 (0.733–1.624)
Always with condom	375 (18.95 %)	66 (31.28 %)	<0.0001	2.2 (1.574–3.054)
*Condom use with casual partners*	589	103	0.7458	
Did not use condom	175 (29.71 %)	32	0.9706	1.01 (0.614–1.659)
Not always with condom	155 (26.32 %)	24	0.4867	0.83 (0.483–1.415)
Always with condom	259 (43.97 %)	47	Ref	1.000
Behavioral characteristics				
*Drug use*	2595	290^b^	<0.001	–
Yes	1844 (71.06 %)	219 (75.52 %)		1.29 (0.973–1.712)
No	751 (28.94 %)	71 (24.48 %)	Ref	
*Illicit drug use*	2591	290^b^	<0.0001	
Yes	471 (18.18 %)	81 (27.93 %)		1.88 (1.421–2.489)
No	2120 (81.82 %)	209 (72.07 %)	Ref	1.000
*Type of Drug consumed*	2606	384^c^	<0.0001	
Alcohol	1811 (69.49 %)	222 (57.81 %)	Ref	1.000
Cannabis	426 (16.35 %)	74 (19.27 %)	<0.0001	38.02 (21.115–68.470)
Cocaine	227 (8.71 %)	39 (10.16 %)	0.0375	1.48 (1.023–2.154)
Crack	134 (5.14 %)	47 (12.24 %)	<0.0001	3.32 (2.283–4.813)
Heroin	1 (0.04 %)	0	0.9701	–
Amphetamines	3 (0.12 %)	0	0.9827	–
Others	4 (0.15 %)	2 (0.52 %)	0.0490	7.19 (1.008–5.133)

a Chi-Square test for Independence.

b Values under the total syphilis population (*n* = 344) due to the lack of data in the medical records.

c The research participants declared the use of more than one type of drug.

Concerning the sociodemographic factors (Table 1), patients aged 20‒29 and 30‒39 years old were the most affected, accounting for more than 60 % of syphilis incidence. Also, marital status accounted for a more significant risk, with those single, divorced, and widowed being more likely to show a positive result for syphilis than married attendees. Moreover, individuals with eight or more years of schooling and healthcare professionals were significantly less likely to have syphilis. On the other hand, there was no statistically significant difference within racial groups. Men were at a higher risk than women; the proportion of syphilis was 57.6 % and 42.4 %, respectively.

Regarding clinical diagnosis (Table 1), patients with a prior clinical history of STI had a 2.36 times higher risk of having syphilis (*p* < 0.0001, OR = 2.36). However, concurrent HIV infection was not a risk factor compared to those without HIV infection. The proportion of HIV-reagent was 10.5 % (*p* < 0.0001, OR = 0.35) for those patients diagnosed with syphilis.

The analysis of sexual behavior (Table 1) showed that patients who self-declared as homosexual were more likely to have syphilis (*p* < 0.0001; OR = 2.93). The proportion of homosexuals was 8.4 % in the study population, but it increased to 18.4 % among those who had syphilis. The proportion of individuals with two or more partners rose from 31.5 % in the study population to 43.8 % among syphilis-positive attendees. Syphilis prevalence was not associated with condom use with steady or casual partners. Although the proportion of individuals who did not use condoms with a steady partner was 51.7 % (*p* = 0.0004), the likelihood of syphilis was higher among those who always used condoms (*p* < 0.001, OR = 2.20).

The use of drugs in the last year was reported by 71.1 % of the total study population and 75.5 % (*p* < 0.0001; OR = 1.29) of those with syphilis. The proportion of illicit drug use was 27.9 % in the syphilis group (*p* < 0.0001; OR = 1.88). Alcohol, cannabis, and cocaine were the most used drugs in the study population, with crack added among those who had syphilis (Table 1).

Among MSM with syphilis, the proportion of individuals with 12 years or more of schooling was 63 %; the proportion of individuals who did not use condoms with a steady partnership was 72 %; of individuals who had multiple sexual partnerships was 67.4 %; and the proportion of drug users was 80.4 %, among whom 21.7 % used illicit drugs. Sixty-three percent of MSM with syphilis had HIV co-infection (*p* = 0.0195; OR = 2.37). This population also showed a 2.84 higher chance of having a previous STI (*p* = 0.00883; OR = 2.84) (Table 2).

**Table 2:** Likelihood ratio test results concerning the significance of variables among Men who have Sex with Men (MSM) with syphilis.

Variables	n ( %)	p-value	OR (95 % CI)
*Schooling (years)*	**46**	0.987	
None	1 (2.2 %)		
1–3	0		
4–7	4 (8.7 %)		
8–11	11 (23.9 %)		
≥12	29 (63.0 %)		
*Multiple partners*	**46**	**0.2379**	
Yes	31 (67.4 %)		
*Condom use with steady partners*	**25**	**0.3257**	
Did not use condom	18 (72.0 %)		
Not always with condom	5 (20.0 %)		
Always with condom	2 (8.0 %)		
*Drug use*	**46**	**0.9343**	
Yes	37 (80.4 %)		
*Illicit drug use*	**46**	**0.5600**	
Yes	10 (21.7 %)		
*HIV co-infection*	**46**	**0.0195**	
Positive	29 (63.0 %)		2.37 (1.122–5.000)
*Previous STI*	**46**	**0.00883**	
Yes	16 (34.8 %)		2.84 (1.300–6.184)

## Discussion

This retrospective cross-sectional observational study included information from 3526 patients who attended in 2018 at the CTC in Goiás, Brazil. We observed higher estimates of syphilis in subgroup populations aged 20‒39, not married, with previous STI, homosexual relationships, high-risk sexual behaviors, and drug use. Our study also provided a comprehensive overview of the syphilis rate in the MSM population. When analyzing this group, we observed that MSM with syphilis had a 2.37 higher risk of HIV infection. Our results are in accordance with some Brazilian and international studies that found similar factors associated with syphilis infection.^14^^,^^15^

In our analysis, the prevalence of syphilis in 2018 (9.8 %) was nearly 20-fold higher than the estimated global syphilis prevalence among women (0.5 %; 95 % Uncertainty Interval [95 % UI 0.4 %–0.6 %]) and men (0.5 %; 0.3–0.7 %) aged 15–49 years in 2012.^16^ Acquired syphilis increased significantly in Brazil in the period from 2011 to 2018. The detection rate increased from 2.1 to 75.8 cases per 100,000 inhabitants between 2014 and 2018, followed by stability, except in 2020, when a decline in the rate was observed, likely due to the COVID-19 pandemic. However, in 2021, the detection rate of acquired syphilis returned to pre-pandemic levels, especially among males and in the age group of 20‒29 years.^17^ Following these data, in our study of 344 syphilis cases, 63 % were between 20 and 39 years old, and 58 % were men.

Our findings indicate syphilis infection was also associated with drug use, the most prevalent illicit substance mentioned to be used by the study population being cannabis. Increases in methamphetamine, injection drug, and heroin use were described in women with syphilis.^18^ Recent data have shown a prevalence of nearly 50 % of substance use in women with a congenital syphilis pregnancy outcome. They were six times and four times as likely to report illicit use of opioids and to report other illegal use of substances during pregnancy, respectively, than those with a noncongenital syphilis pregnancy outcome.^19^

When analyzing our data in the MSM group, we can deduce that syphilis augments the risk of transmitting or acquiring HIV and other STIs. A wide range of studies report an increase in the rates of syphilis among homosexuals, mainly MSM, and that syphilis infection risk increases with high-risk sexual behaviors.^20^, ^21^, ^22^, ^23^ Our study supports the need for strategies of continued engagement and high priority of prevention, screening, and treatment of syphilis infection at the regional level in this MSM group.

The likelihood of having syphilis was high among patients who reported consistently using condoms during sexual relationships. This bias could be due to the fact that some attendees may feel embarrassed to report their sexual behavior or to mention the STI history of their sexual partnerships to the healthcare professional during the consultation and advisory.

Our study has some limitations. Firstly, behavioral aspects linked to sexual and social characters are susceptible to subjective memory bias. A second limitation is that our study population was restricted to a convenience sample from a single CTC and may not be generalized to other populations. However, the CTC in Goiás is the only STI testing and referral center in the micro-region studied, and the positive data is representative of all public sector services related to this condition. To minimize bias in data collection, we used information obtained from the CTC Information System (SICTC) records, which were collected by a healthcare professional specialized in sexual counseling. The data was collected through interviews in two distinct moments to achieve greater collaboration and truthfulness in behavioral reports, given the relevance of social and sexual aspects in identifying vulnerable populations.

The reasons for the resurgence of syphilis are multifactorial and complex.^8^ Unfortunately, the Brazilian Health Information System (SINAN) does not address issues related to sexual and social behaviors in the mandatory notification, which are crucial in the context of syphilis. Based on the data presented here, we suggest including information about social and sexual determinants to improve future strategies for notification, management, prevention, and treatment of syphilis. This can allow for the implementation of effective strategies for vulnerable groups to effectively reach key populations, aiming to reduce the transmission of STIs and syphilis in the general population.

This study’s cross-sectional design enables the identification of associations between sociodemographic and behavioral factors and syphilis infection among people living with HIV. However, it does not allow for conclusions regarding the temporal sequence or causality of these relationships. As such, caution is warranted when interpreting these findings, and further longitudinal studies are needed to explore potential causal pathways. Our data provide a valuable referral benchmark by establishing a foundation for evaluating changes over time, so as to guide or provide grounds for future assessments.

## Conclusion

Syphilis detection among patients who attended the CTC in Goiás, Brazil, is unacceptably high, especially among young adults, drug users, MSM, and individuals with previous STIs. These findings can support the implementation of public health strategies to prevent, screen, control, and treat this chronic multistage disease, especially for high-risk populations, aiding in the pursuit of the goal of reducing syphilis incidence worldwide by 90 % by 2030.

## Funding

This work was supported by grants awarded by the Fundação de Amparo à Pesquisa do Distrito Federal (FAP-DF, grant 0193.00002600/2022–70) and by the 10.13039/501100002322Coordenação de Aperfeiçoamento de Pessoal de Nível Superior (CAPES, grant 88881.711954/2022-01 CAPES-COFECUB).

## Conflicts of interest

The authors declare the following financial interests/personal relationships which may be considered as potential competing interests:

Cristhiane Campos Marques and Berenice Moreira are employees at the Centro de Testagem e Aconselhamento in Rio Verde, Goiás, Brazil.

Otávio de Toledo Nóbrega declares to be supported by a fellowship in research productivity by CNPq.

The remaining authors declare that they have no known competing financial interests or personal relationships that could have appeared to influence the work reported in this paper.
